# Effect of Filler Content on the Morphology and Physical Properties of Poly(Lactic Acid)-Hydroxyapatite Composites

**DOI:** 10.3390/ma16020809

**Published:** 2023-01-13

**Authors:** Nedjma Tazibt, Mustapha Kaci, Nadjet Dehouche, Mohamed Ragoubi, Leonard Ionut Atanase

**Affiliations:** 1 Laboratoire des Matériaux Polymères Avancés, Faculté de Technologie, Université de Bejaia, Bejaia 06000, Algeria; 2 UnilaSalle, Unité de Recherche Transformation et Agro-Ressources, VAM^2^IN (EA 7519 UniLaSalle—Université d’Artois), F-76130 Mont-Saint-Aignan, France; 3 Faculty of Medical Dentistry, “Apollonia” University of Iasi, 700511 Iasi, Romania; 4 Academy of Romanian Scientists, 050045 Bucharest, Romania

**Keywords:** poly(lactic acid), hydroxyapatite, biocomposites, morphology, mechanical properties

## Abstract

The effect of hydroxyapatite (HAp) synthesized by the chemical precipitation process on the morphology and properties of composites based on poly(lactic acid) (PLA) was investigated at various filler content ratios, i.e., 5, 10 and 15 wt%. Both neat PLA and PLA-based composites were first prepared using the solvent casting method, followed by melt compounding in an internal mixer, whereas tensile specimens were obtained by thermo-compression. The study revealed that the addition of 5 wt% of HAp into the PLA led to a slight improvement in both the thermal stability and tensile properties of the composite material in comparison with neat PLA and other composite samples. Indeed, the values of the tensile strength and modulus increased from approximately 61 MPa and 2.9 GPa for the neat PLA to almost 64 MPa and 3.057 GPa for the composite sample, respectively. Moreover, the degradation temperature at a 5 wt% mass loss also increased by almost 5 °C compared to other samples, due probably to a finer dispersion of the HAp particles in the PLA, as observed under a scanning electron microscope. Furthermore, the FT-IR spectra displayed some changes in the chemical structure of the PLA/HAp (5 wt%), indicating the occurrence of filler-matrix interactions. At a higher filler content ratio, a decrease in the properties of the PLA/HAp composites was observed, being more pronounced at 15 wt%. The PLA composite containing 5 wt% HAp presents the best compromise among the investigated properties. The study highlighted the possibility of using HAp without any prior surface treatment as a reinforcement in PLA composite materials.

## 1. Introduction

Biodegradable polymers have attracted the attention of academics and the industry over the past two decades due to the growing crisis of environmental pollution. These environmentally friendly materials have been recognized as a promising replacement for polymers derived from fossil petroleum, thereby reducing waste pollution due to their biodegradability [1]. Among the family of biodegradable polymers, poly(lactic acid) (PLA) is a bio-based polymer made from renewable resources, such as the starch from corn and potatoes, maize and sugar cane, etc. The carbon in PLA comes from the carbon dioxide in the atmosphere, which is immobilized in glucose through photosynthesis; therefore, carbon dioxide produced through its removal, incineration or biodegradation does not increase the total amount of carbon dioxide in the atmosphere [2]. The biocompatibility, bioresorbability and biodegradability of PLA [3] recommend this polymer as being suitable for biomedical and food packaging, bone tissue engineering, 3D-printed scaffold fabrication and surgical suturing [4], besides other applications including drug carrier agents [5,6], controlled-release drugs and orthopedic implants [7].

Although PLA has many good properties, its low tenacity and slow crystallization speed limit its use in other industrial applications requiring a higher mechanical resistance [8]. To overcome these drawbacks, one of the most common approaches consists of adding either organic or inorganic fillers to the PLA, including organo-modified layered silicates [9], hydroxyapatite [10], talc [11], cellulose [12], carbon nanotubes, metallic oxides and others to improve the functional characteristics of the materials [13,14,15,16].

In this regard, hydroxyapatite (HAp), having the following chemical formula, Ca_10_(OH)_2_(PO_4_)_6_), is considered as a reliable reinforcing bio-filler for many polymers [17,18,19,20,21]. HAp is ranked as bioactive, non-toxic, osteoinductive and osteoconductive ceramic of great significance for bone scaffolds, due to the similar properties with the mineral portion of bones and teeth and its ability to form direct chemical bonds with living tissue [22]. Thus, reinforcing PLA with HAp could sufficiently combine their respective advantages. This combination may help to improve the mechanical strength, rate of disintegration of PLA, thermal stability and rheological properties of PLA [23]. Furthermore, PLA/HAp composites have a good biocompatibility and exhibit a structure similar to that of natural bones, and therefore they can induce easily osteogenesis.

There are many studies available in the literature on the synthesis methods as well as the structure–properties relationships of polymer/HAp composites [24,25]. One of them concerned PLA/HAp composites which were prepared by solution intercalation method. The formation of an exfoliated structure with a considerable improvement of static and dynamic mechanical properties was observed, in particular at an optimum filler content of 20 wt% [26]. Abu Bakar et al. [27] reported the processing steps involved in the preparation of HAp and semicrystalline polyether-ether-ketone (PEEK) composites elaborated by the injection molding process at various filler contents and their effect on the mechanical properties. The authors showed the feasibility of manufacturing PEEK/HAp composites up to 40 vol% of HAp. Tensile and micro-hardness increased with the amount of HAp. Jaafar et al. [28] reported that the tensile and flexural properties of HDPE/HAp composites depend on the filler content. Indeed, by increasing the HAp content up to 30 wt%, the tensile strength, flexural strength and impact strength of the composite materials decreased. Moreover, the authors indicated that the major constraint encountered in the preparation of the HDPE/HAp composites by melt compounding, lies in the difficulty to disperse HAp in the matrix due to filler aggregates formation [29]. However, the silane-treated composites showed better interactions between HDPE and HAp due to the elimination of some polar groups of HAp, i.e., C=O and silanol groups leading to a less hydrophilic filler. Lin et al. [30] reported that by varying the HAp content ratio from 0 to 40 wt% in PLA composites prepared by solvent casting, the mechanical strength and cellular proliferation of materials were affected. The study allowed the optimizing of the properties of PLA composites, which can be further used for different medical applications. Despite the numerous papers published on polymer-HAp composites, further work is required, however, to be done for the development of new performing PLA/HAp materials. Indeed, from the literature review, it appears that for the melt blending of PLA/HAp composites, most publications focused on the surface modification of HAp, the functionalization of the PLA or the addition of an interfacial agent to improve the filler–matrix adhesion. However, there were few works concerning the property tunability of PLA/HAp without any prior chemical modification.

Therefore, this work aims at studying the structure–properties relationships of PLA/HAp composites, prepared using the solvent casting method and melt compounding process at various HAp content ratios, i.e., 5, 10 and 15 wt%. The effect of filler content on the PLA composites was investigated with several techniques, involving scanning electron microscopy (SEM), Fourier transform infrared spectroscopy (FT-IR), X-ray diffraction (XRD), thermogravimetric analysis (TGA) and tensile measurements with respect to neat PLA. Moreover, the paper also describes the route to synthesize HAp powder using the chemical precipitation technique, and subsequently, the characterization study of the product in terms of morphology, crystallinity, chemical composition and particle size distribution.

## 2. Materials and Methods

### 2.1. Materials

Ca(NO_3_)_2_·4H_2_O, (NH_4_)_2_HPO_4_ and Chloroform (CHCl_3_) were purchased from BIOCHEM Chemopharma (Cosne-Cours-sur-Loire, France), while (NH_4_OH) was purchased from (Alfa Aesar, Haverhill, MA, USA). PLA 7001D was supplied in pellet form by Nature Works (Plymouth, MN, USA). The polymer is semi-crystalline with the following properties: density = 1.25 g/cm^3^, MFI = 6 g/10 min (210 °C, 2.16 kg), Tg = 60 °C and Tm = 160 °C.

### 2.2. HAp Synthesis

Hydroxyapatite (HAp) was prepared in a powder form under atmospheric conditions by using the solution–precipitation method [31], which involves calcium nitrate as the only source of calcium ions, ammonium phosphate dibasic as the only source of phosphate and ammonia solution for pH adjustment. A total of 0.06 M of di-ammonium hydrogen phosphate solution and 0.1 M of calcium nitrate tetrahydrate solution were prepared, and the pH of both solutions was maintained at 11 by adding a small amount of aqueous ammonia. The phosphate solution was drip-fed into the calcium nitrate solution, resulting in HAp precipitation. The purification step was carried out by washing the precipitate, which was aged during the night, with demineralized water. The obtained product was subsequently air-dried, weighed and analyzed using several techniques in terms of crystallinity, morphology and elemental composition. The basic chemical reaction involved in the synthesis of HAp powder is well described in Equation (1) [28]:

10Ca(NO_3_)_2_ + 6(NH_4_)_2_HPO_4_ + 8NH_4_OH → Ca_10_(PO_4_)_6_(OH)_2_ + 20NH_4_NO_3_ + 20H_2_O (1)

### 2.3. Preparation of PLA/HAp Composites

To ensure a good dispersion of HAp in the polymer matrix before melt compounding, a solvent casting method was used to prepare the thin film samples according to the following procedure: Prior, PLA pellets and HAp powder were dried in an oven at 70 °C for 24 h. Then, 2 g of PLA was dissolved in 20 mL of chloroform at room temperature for 3 h under magnetic stirring. In parallel, HAp powder at various content ratios, i.e., 5, 10 and 15 wt%, with respect to neat PLA, was dispersed into a chloroform solution under the same stirring conditions and time. After this, the two solutions were mixed and stirred for 3 more hours. Finally, the solutions of PLA/HAp were poured into Petri dishes and dried overnight. The cast films obtained were cut into flakes for melt mixing. For comparison, PLA films were prepared in the same experimental conditions. Prior to melt blending, PLA and various PLA/HAp composite flakes were vacuum dried at 60 °C for 24 h to avoid any hydrolysis of PLA. All materials were subjected to melt compounding in a Brabender Plasticorder internal mixer (model W 50 EHT) (New Jersey, USA) at 5, 10 and 15 wt% (Table 1), using the following processing conditions: mixing temperature = 180 °C, screw speed = 50 rpm and residence time = 8 min.

### 2.4. Characterization

FT-IR spectroscopy was used to investigate any changes in the chemical structure of the samples using a Shimadzu (Tokyo, Japan) IR Prestige-21 FT-IR spectrometer with the KBr pellet method. FT-IR spectra of both the synthesized HAp, neat PLA and various PLA composite samples were recorded in a spectral range from 4000 to 400 cm^−1^ at a 4 cm^−1^ resolution and with 40 scans.

XRD analysis was performed using a PAnalytical EMPREAN diffractometer with CuKα radiation (wavelength λ = 0.154 nm) generated at 40 kV and 200 mA. The acquisition rate was 1°/min for a range from 10–80°. Thermal stability was determined using a TGA (PerkinElmer, Waltham, MA, USA) analyzer from ambient temperature to 1000 °C at a heating rate of 10 °C/min under a nitrogen atmosphere; an average mass of 10 mg of each sample was used.

Elemental analysis and morphology of the samples were investigated using an EDS (JEOL EDS SYSTEM, Tokyo, Japan)-equipped electron microscope. The neck region of the samples fractured with liquid nitrogen is parallel to the extraction direction to reveal the internal morphology. Before the observation, the fracture surfaces were covered in a thin layer of gold using a Polaron sprayer.

Size distribution of HAp powders was investigated by a particles size analyzer (MALVERN Hydro 2000 MU (A), Malvern, UK). For the measurements, 2 g of powder were dispersed into 100 mL of distilled water, in an ultrasound bath, for 3 min.

Tensile measurements were performed on both neat PLA and PLA composites samples using an MTS Systems machine according to ISO 527. The specimens with dimensions of 75 × 5 × 2 mm (ISO 527-2-1BA) were tested in tension rate of 1 mm/min at room temperature (23 °C) and 45% of relative humidity. Five replicates were performed on each formulation sample obtained using compression molding. Notched IZOD impact test was performed by using a RESIL IMPACTOR CEAT model 46/000 on specimens (Cerreto d’Esi (AN), Italy) having the following dimensions: 63 × 12.7 × 2 mm according to ASTM D-256-73. Four replicates were tested.

## 3. Results and Discussion

### 3.1. Characterization of the Chemical Structure, Composition, Morphology and Physical Properties of Synthesized HAp

#### 3.1.1. Chemical Structure Analysis by FT-IR Spectroscopy

**Figure 1:**
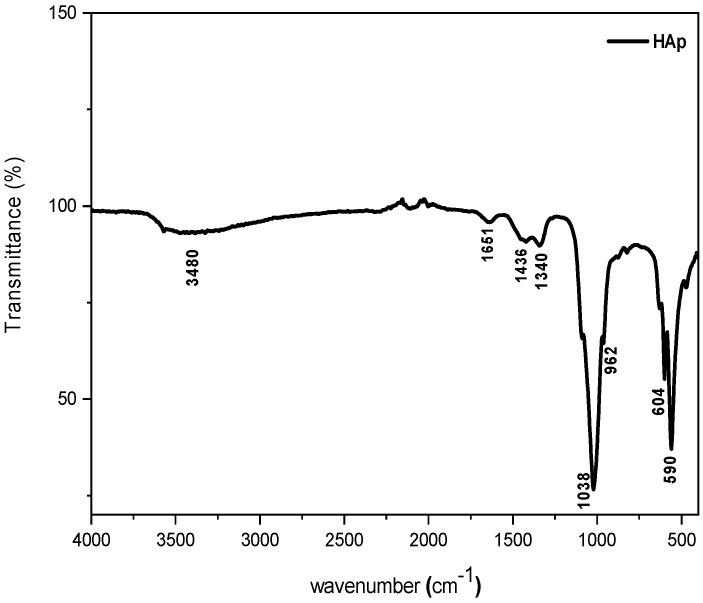
Typical FT-IR spectrum of the synthesized HAp.

FT-IR spectroscopy was used in order to investigate the chemical structure of the synthesized HAp powder after drying the sample at 80 °C overnight. The corresponding FT-IR spectrum is illustrated in Figure 1, on which it can be observed the occurrence of two absorption bands located at 1038 and 962 cm^−1^, corresponding to stretching vibrations of phosphate groups PO_4_^3−^ in HAp [32]. Further, two other peaks are observed at 590 and 604 cm^−1^, attributed to the deformation vibration of PO_4_^3−^ groups [33]. The absorption band centered at 1651 cm^−1^ and the large band at 3480 cm^−1^, both of which have a weak intensity, correspond to the H_2_O deformation [34]. There are also some other small peaks at 1340 and 1436 cm^−1^, which are assigned to CO_3_^2−^ ions, resulting probably from CO_2_ absorption on the surface of HAp particles [35]. Regarding the above results, the synthesized HAp is a pure substance [33].

#### 3.1.2. Crystallinity Analysis of HAp by XRD

**Figure 2:**
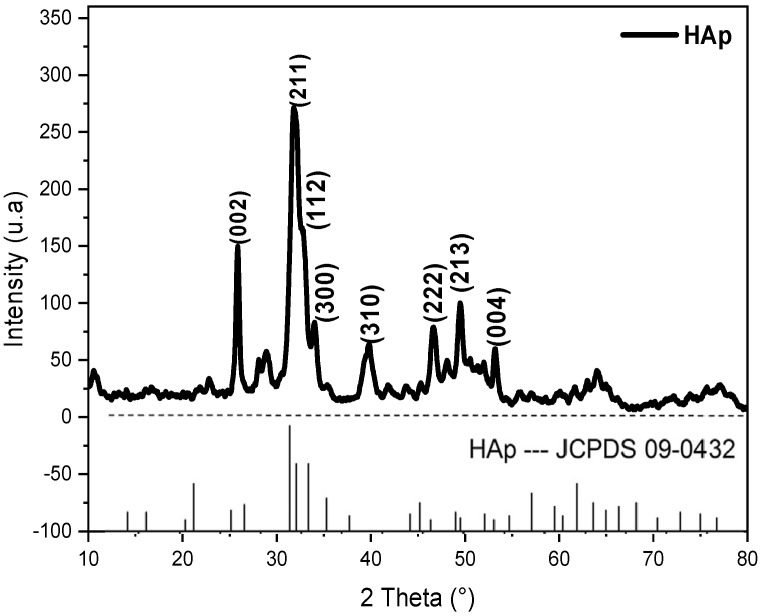
X-ray diffraction pattern of the synthesized HAp particles.

Figure 2 shows the XRD pattern of the synthesized HAp, which is indexed to the standard JCPDS (Joint Committee on Powder Diffraction Standards) data of the HAp particle (JCPDS 09-0432). The corresponding characteristic peaks observed at 25°, 31°, 32°, 33°, 39°, 46°, 49° and 53° are attributed to (002), (211), (112), (300), (310), (222), (213) and (004) planes, respectively [34,36]. The peaks are in good correlation with standard hydroxyapatite, namely Ca_10_(PO_4_)_6_(OH)_2_, and the diffraction model corresponds well to the hexagonal structure of HAp [31].

From Figure 2, the XRD pattern of the synthesized HAp exhibits a pure structure and seems to not contain any traces of calcium phosphate impurities [37].

#### 3.1.3. Thermal Stability by TGA

**Figure 3:**
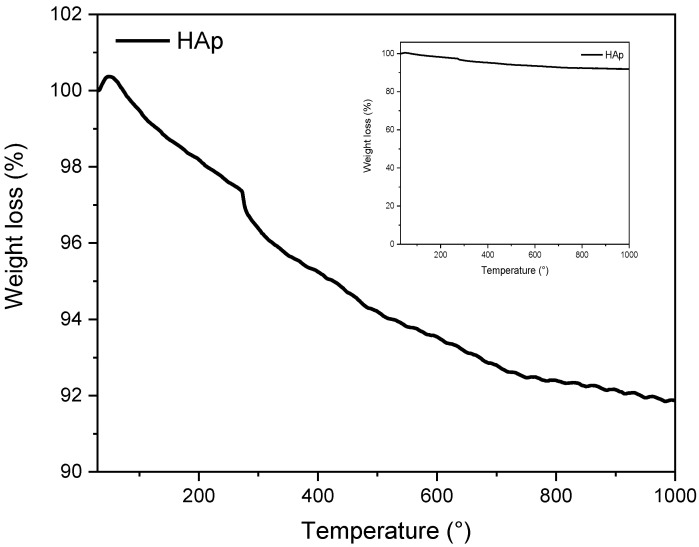
TGA thermogram of the synthesized HAp.

TGA thermogram of the synthesized HAp is illustrated in Figure 3 and consists of three degradation steps. In the first step, up to 250 °C, there is a loss of water molecules absorbed at the surface of the HAp powder [38]. The second step, the thermal degradation of the synthesized HAp occurs in the temperature range between 250 and 600 °C, resulting from the vaporization of the HAp crystallization water. In the last step, starting from 600 to 1000 °C, the decrease in weight loss is slowed down and only 2 wt% were noted, which is attributed to the breakage of CO_3_^2−^ and HPO_4_ ions in the HAp [19]. Moreover, the TGA thermogram shows a little weight loss of approximately 8 wt%, clearly indicating that the synthesized HAp is thermally stable up to 1000 °C.

#### 3.1.4. Energy Dispersive X-ray Analysis (EDX)

**Figure 4:**
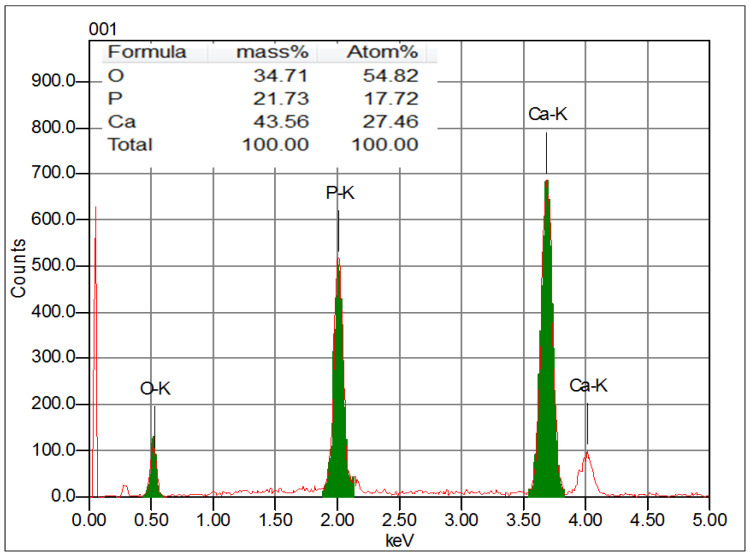
EDX spectrum of the synthesized HAp powder.

In order to determine the chemical composition of the synthesized HAp, the energy dispersive X-ray analysis was used. The data are presented in Figure 4. Accordingly, the composition of the sample is based on the following chemical elements, i.e., Ca, P and O, which are the constituents of HAp. No other chemical elements are detected. This result clearly confirms the high purity of the synthesized HAp powder, which agrees with the literature data [39].

#### 3.1.5. Morphological Characterization of HAp by SEM and Particle Size Distribution

**Figure 5:**
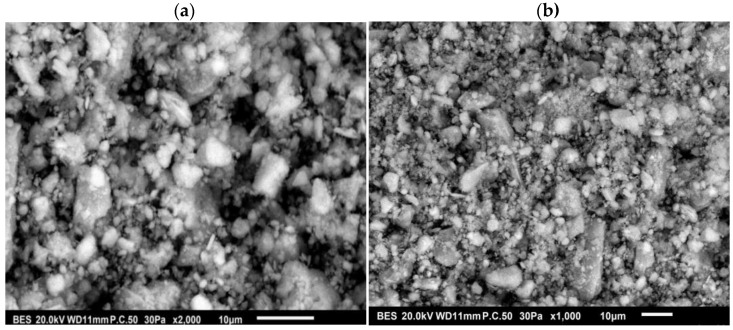
SEM micrographs of the synthesized HAp at (**a**): ×2000 and (**b**): ×1000.

**Figure 6:**
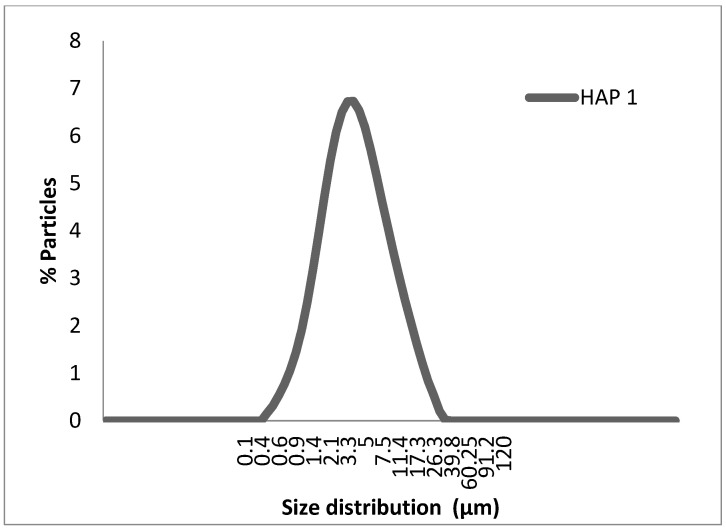
Plot of particle size distribution of the synthesized HAp powder.

SEM micrographs of HAp particles are illustrated in Figure 5a,b corresponding to 2000 and 1000 magnifications, respectively. Figure 5a,b clearly shows that the shape of the particles is mostly spherical, but some particles are less regularly shaped. Furthermore, the particles exhibit a wider size distribution. Indeed, the synthesized HAp powder forms aggregates. Figure 6 depicts the plot of the particle fraction as a function of the particle size distribution, and it appears that the average size distribution is between 0.4 and 8 μm. This is consistent with the data provided by Kesmez et al. [40], who indicated the formation of wide agglomerated particles having an average particle size ranging from 1 to 15 μm.

### 3.2. Effect of Filler Content on Morphology and Properties of PLA/HAp Composites

#### 3.2.1. FT-IR Analysis

**Figure 7:**
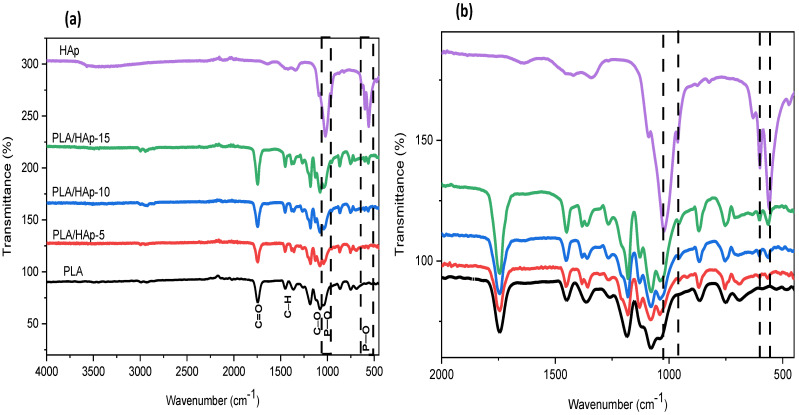
FT-IR spectra of neat PLA and PLA/HAp composites at various filler content, i.e., 5, 10 and 15 wt%, recorded in the regions: (**a**): 4000–400 cm^−1^ and (**b**): 2000–500 cm^−1^.

FT-IR spectra of neat PLA and PLA/HAp composites at 5, 10 and 15 wt% and recorded in the range 4000–500 cm^−1^ are shown in Figure 7. In the spectrum of neat PLA, the absorption bands observed at λ_max_ = 2998 and 2947 cm^−1^ are assigned to the asymmetrical and symmetrical stretching vibrations of C–H bonds, respectively. The absorption band centered at λ_max_ = 1751 cm^−1^ is attributed to the stretching vibrations of the ester bond and the absorption band at 1466 cm^−1^ is due to the deformation vibration of the CH_3_ group. Moreover, the absorption bands located at 1183 cm^−1^ and 1084 cm^−1^ correspond to the binding vibrations of C–O–C ether bonds. Another band is also observed at 873 cm^−1^ corresponding to the C–COO bond vibrations. Similar observations were previously reported in the literature [41,42].

FT-IR spectra of PLA/HAp composites display the characteristic absorption bands of both PLA and HAp; the intensity depends strongly on the blend composition. Indeed, it is observed that the intensity of the absorption bands at 1038, 604, 962 and 562 cm^−1^, corresponding to the PO_4_^3−^ vibration in HAp, increases with the filler content. Based on the absorption band located at 1084 cm^−1^ (νC-O-C), which remains unchanged in all samples, thus taken as reference one for normalization, changes in the band intensity at 1038 cm^−1^ are clearly observed in the composite samples compared to neat PLA, which may indicate the plausible occurrence of some interactions between HAp and the polymer chains [5].

#### 3.2.2. XRD Analysis

**Figure 8:**
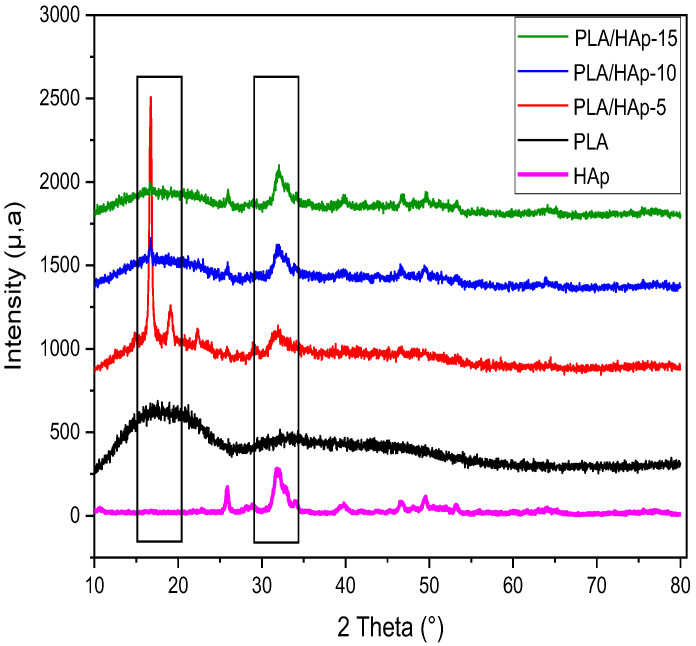
XRD patterns of HAp, PLA and PLA/HAp composites at various filler content ratios, i.e., 5, 10 and 15 wt%.

Figure 8 shows the XRD patterns for neat PLA, HAp powder and PLA/HAp composites at various filler content ratios, i.e., 5, 10 and 15 wt%. The XRD pattern of PLA shows a broad band with a maximum at 2θ = 16.5°, which indicates a completely amorphous structure [43]. For PLA/HAp composites, the XRD pattern exhibits some characteristic peaks of HAp powder at approximately 25°, 31° and 33°, which are assigned to the (002), (211) and (300) reflections, respectively. Furthermore, there is also an increase in the peak intensity with increasing the HAp content, as reported in the literature [44]. In Figure 8, the XRD spectrum of the PLA/HAp composite, loaded at 5 wt%, displays two sharp diffraction peaks, the first one is located at 2θ = 16.6° and the other at 19.8°, which correspond respectively to the plans (110)/(200) and (203)/(113). According to the literature [16,45], this indicates the growth of new crystals in the PLA matrix promoted by the HAp particles acting as a heterogeneous nucleating agent [45]. However, at 10 and 15 wt% filler content, this phenomenon is not observed in the PLA composites, due probably to the presence of filler aggregates which disturb the crystallization growth.

#### 3.2.3. Thermal Stability by TGA

**Figure 9:**
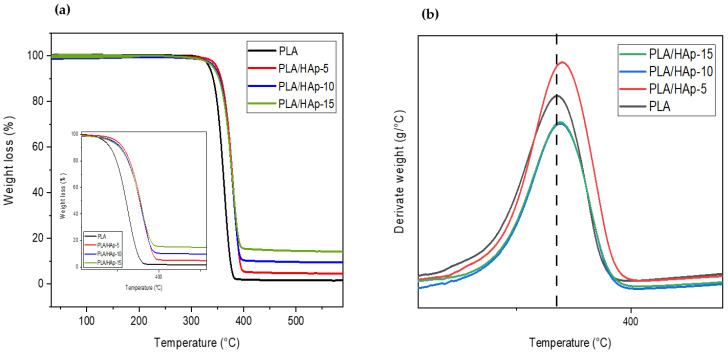
TGA (**a**) and DTG (**b**) thermograms for neat PLA and PLA/HAp composites at the filler content ratios of 5, 10 and 15 wt%.

The effect of the HAp content on the thermal stability of the PLA composites was evaluated by TGA. In this regard, Figure 9a,b show, respectively, the TGA and DTG thermograms of neat PLA and PLA/HAp composites, filled at 5, 10 and 15 wt%. Figure 9a displays all TGA thermograms of the composite samples showing one degradation step corresponding to the thermal degradation of PLA. Figure 9b displays the DTG thermograms, where a slight increase in both T_5_ (decomposition temperature at 5 wt% mass loss) and T_mdr_ (temperature at maximum degradation rate) is observed at a 5 wt% filler content, compared to neat PLA and other composite samples. Table 2 summarizes the TGA data for both neat PLA and PLA composites. From Table 2, the incorporation of HAp at a filler content of 10 and 15 wt% has almost no effect on the thermal stability of the composite materials compared to the neat PLA. This is due probably to the poor dispersion of HAp in the polymer matrix, as reported in the literature [23]. Indeed, the tendency of the HAp powder to form aggregates in the PLA matrix at a higher content does not contribute to thermal stability of the composite materials. Conversely, the increase in thermal stability in PLA/HAp (5 wt%) may result from the homogeneous and finer dispersion of the filler in the PLA and subsequently, an enhancement in filler–matrix interactions [46]. Indeed, the good dispersion of HAp may slow down PLA decomposition products and act as a barrier. In this regard, Yang et al. [47] reported that the addition of 10 wt% of montmorillonite (MMT) in polyimide composite decreases the thermal stability of the composite material due to the poor dispersion of MMT in the polymer matrix, whereas at 5 wt%, the thermal stability is improved due to a better dispersion of the clay into the polyimide matrix.

#### 3.2.4. Mechanical Properties of PLA/HAp Composites

**Figure 10:**
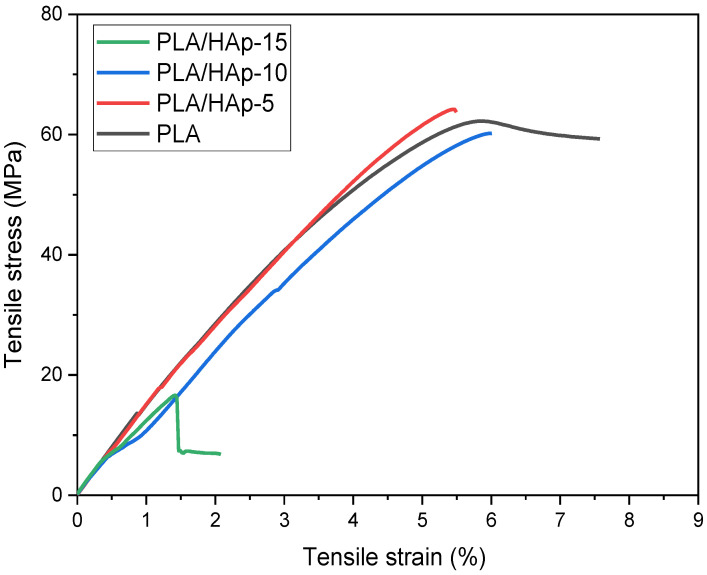
Representative stress–strain curve of the neat PLA and PLA/HAp composites at various filler contents, i.e., 5, 10 and 15 wt%.

**Figure 11:**
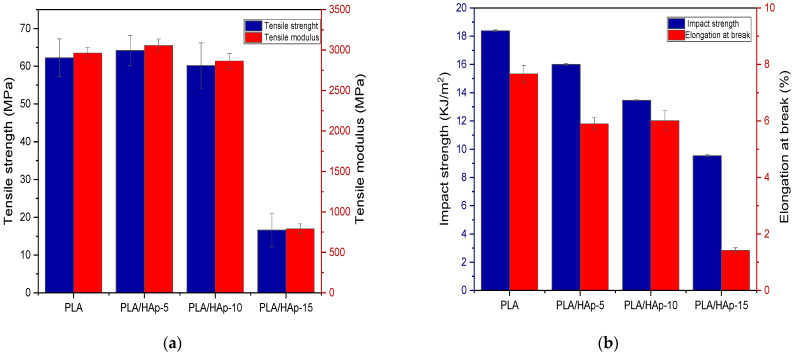
Tensile strength and tensile modulus (**a**) and impact strength and elongation at break (**b**) of the neat PLA and PLA/HAp composites at various filler content ratios, i.e., 5, 10 and 15 wt%.

Figure 10 shows the stress–strain curves of neat PLA and PLA/HAp composites. As expected, the shape of the curves is characteristic of brittle materials. Indeed, both PLA and HAp are rigid and brittle. In more details, Figure 11a shows the histograms of tensile strength and elastic modulus, while Figure 11b displays those of impact strength and elongation at break for the neat PLA and PLA-based composites. It can be seen in Figure 11a that both the tensile strength and elastic modulus of PLA/HAp (5 wt%) have slightly increased than those of the neat PLA, even more than those of other composites. Indeed, the values of the elastic modulus and tensile strength of PLA/HAp (5 wt%) are 3057 and 64 MPa, respectively, while for the neat PLA, the values are 2963 and 62 MPa, respectively. A good dispersion of HAp in the polymer matrix coupled with the stiff character of the filler may be responsible for the increase in modulus in the filled sample at 5 wt% [46]. However, above 5 wt%, there is a decrease in both the tensile characteristics of the PLA composites, being more pronounced at 15 wt%. The formation of the filler aggregates weakens the stress transfer between the PLA matrix and HAp, acting as stress concentrators. In fact, filling aggregates disturb the continuity of the matrix and limit the capacity of the polymer chains to withstand stress. Figure 11b shows the histograms of impact strength and the elongation at the break of PLA composites in comparison with neat PLA. It is clearly observed a gradual decrease in both properties with increasing the filler content. The large agglomeration of HAp particles in the PLA matrix reduces the contact area and creates physical defects in the composite samples [26]. Moreover, the incorporation of HAp into PLA limits both the movement of the polymer chains and their deformation [15].

**Figure 12:**
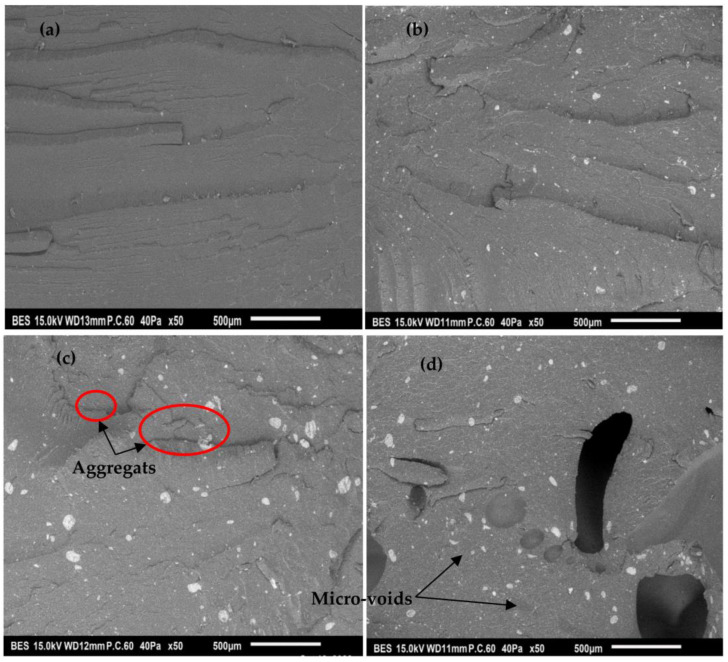
SEM images of the fractured surfaces after impact testing of: PLA (**a**), PLA/HAp-5 (**b**), PLA/HAp-10 (**c**) and PLA/HAp-15 (**d**); at 50×.

Figure 12 shows the presence of HAp aggregates on the fracture surface of PLA/HAp composites subjected to an impact test. This may be responsible for the decrease in tensile strength. This result agrees with the literature data [48].

#### 3.2.5. SEM Analysis

For a better comprehension of the relationships between the morphology of the PLA/HAp composites and the mechanical behavior, SEM micrographs are shown in Figure 12a–d, corresponding to the fracture surface of the neat PLA and PLA composites at 5, 10 and 15 wt%, respectively. Figure 12a, relative to the PLA matrix, exhibits a relatively smooth surface, exhibiting no defects. Figure 12b shows the fracture surface of the PLA filled at 5 wt%. The fracture surface shows a finer and homogeneous dispersion of HAp particles in the PLA matrix compared with the 10 and 15 wt% filled composites illustrated in Figure 12c,d, respectively. It clearly appears that the formation of HAp aggregates is more enhanced at a higher content, i.e., 10 and 15 wt% rather than at 5 wt%. This does not mean that no aggregates are formed in the PLA/HAp at 5 wt%, but that the number of aggregates and their size are less important than others. This is shown in Figure 12b where HAp microparticles (within a few microns in size) are finely dispersed on the fracture surface of PLA. Whereas in Figure 12b,c, it can be seen that the number as well as size aggregates have increased markedly resulting in a rough surface [49]. Furthermore, a gap at the HAp–PLA interface appears clearly on the impact fracture surface of the PLA-based composite at 15 wt%, as illustrated in Figure 12d. Consequently, HAp load aggregation in the PLA composites could be responsible for the reduction of the mechanical properties. Indeed, filler aggregates can serve as stress concentration points that can lead to the premature failure of the composites [50].

## 4. Conclusions

HAp was synthesized in the laboratory using the chemical precipitation method and the product was characterized. Indeed, the chemical structure, crystallinity, elemental chemical composition, morphology and size distribution showed that the synthesized HAp powder is pure and free of contaminants.

The effect of the HAp content on the morphology and properties of the PLA/HAp composites, prepared by combining both the solvent casting method and melt compounding, was also evaluated at 5, 10 and 15 wt%. Regarding the whole results, the PLA composite containing 5 wt% HAp presents the best compromise among the investigated properties, due probably to the finer dispersion of the filler in the polymer matrix. However, at a filler content of 10 and 15 wt%, the PLA/HAp composites exhibit lower mechanical properties. The formation of the HAp aggregates in the PLA matrix, as observed by SEM could be responsible for the weakness of the tensile properties, as well as the impact strength. The study highlights the role of HAp as a reinforcement in PLA composites at 5 wt% without any prior chemical modification of the filler.

## Figures and Tables

**Table 1:** Code and composition of the various formulas elaborated by melt compounding.

Samples	Compositions	
	PLA (wt%)	HAp (wt%)
PLA	100	0
PLA/HAp-5	95	5
PLA/HAp-10	90	10
PLA/HAp-15	85	15

**Table 2:** Values of T_5_, T_mdr_ and (%) residue at 600 °C of neat PLA and PLA/HAp composites.

Samples	T_5%_ (°C)	T_mdr_ (°C)	Residues (%) at 600 °C
PLA	334 ± 0.5	363 ± 0.6	1.6 ± 0.1
PLA/HAp-5	340 ± 0.9	371 ± 0.8	4.6 ± 0.1
PLA/HAp-10	335 ± 0.6	367 ± 0.5	9.5 ± 0.2
PLA/HAp-15	333 ± 0.7	365 ± 0.4	14.2 ± 0.5
